# Prevalence of depression and anxiety among participants with glaucoma in a population-based cohort study: The Gutenberg Health Study

**DOI:** 10.1186/s12886-018-0831-1

**Published:** 2018-06-28

**Authors:** J. Rezapour, S. Nickels, A. K. Schuster, M. Michal, T. Münzel, P. S. Wild, I. Schmidtmann, K. Lackner, A. Schulz, N. Pfeiffer, M. E. Beutel

**Affiliations:** 1 Department of Ophthalmology, University Medical Center Mainz, Mainz, Germany; 2 Department of Psychosomatic Medicine and Psychotherapy, University Medical Center Mainz, Mainz, Germany; 3 Center for Cardiology I, University Medical Center Mainz, Mainz, Germany; 4 Preventive Cardiology and Preventive Medicine / Center for Cardiology, University Medical Center Mainz, Mainz, Germany; 5 Center for Thrombosis and Hemostasis (CTH), University Medical Center Mainz, Mainz, Germany; 6 German Center for Cardiovascular Research (DZHK), partner site Rhine-Main, Mainz, Germany; 7 Institute for Medical Biostatistics, Epidemiology and Informatics, University Medical Center Mainz, Mainz, Germany; 8 Institute for Clinical Chemistry and Laboratory Medicine, University Medical Center Mainz, Mainz, Germany

**Keywords:** Glaucoma, Depression, Anxiety, Prevalence, Population-based cohort study, European cohort

## Abstract

**Background:**

To investigate the prevalence of depression and anxiety among subjects with self-reported glaucoma and the association between self-reported glaucoma and depression respectively anxiety in a European cohort.

**Methods:**

A study sample of 14,657 participants aged 35 to 74 years was investigated in a population-based cohort study. All participants reported presence or absence of glaucoma. Ophthalmological examinations were carried out in all participants and demographic and disease related information were obtained by interview. Depression was assessed with the Patient Health Questionnaire (PHQ-9), and generalized anxiety with the two screening items (GAD-2) of the short form of the GAD-7 (Generalized Anxiety Disorder-7 Scale). Prevalence of depression and generalized anxiety were investigated for subjects with and without self-reported glaucoma. Logistic regression analyses with depression, respectively anxiety as dependent variable and self-reported glaucoma as independent variable were conducted and adjusted for socio-demographic factors, systemic comorbidities (arterial hypertension, myocardial infarction, stroke, diabetes mellitus, chronic obstructive pulmonary disease, cancer), ocular diseases (cataract, macular degeneration, corneal diseases, diabetic retinopathy), visual acuity, intraocular pressure, antiglaucoma eye drops (sympathomimetics, parasympathomimetics, carbonic anhydrase inhibitors, beta-blockers, prostaglandins) and general health status.

**Results:**

293 participants (49.5% female) reported having glaucoma. Prevalence of depression among participants with and without self-reported glaucoma was 6.6% (95%-CI 4.1–10.3) respectively 7.7% (95%-CI 7.3–8.2), and for anxiety 5.3% (95%-CI 3.1–8.7) respectively 6.6% (95%-CI 6.2–7.1). Glaucoma was not associated with depression (Odds ratio 1.10, 95%-CI 0.50–2.38, p = 0.80) or anxiety (1.48, 95%-CI 0.63–3.30, p = 0.35) after adjustment for socio-demographic factors, ocular/systemic diseases, ocular parameters, antiglaucoma drugs and general health status. A restriction to self-reported glaucoma cases either taking topical antiglaucoma medications or having a history of glaucoma surgery did not alter the result.

**Conclusions:**

This is the first study analyzing both depression and anxiety among glaucoma patients in a European cohort. Subjects with and without self-reported glaucoma had a similar prevalence of depression and anxiety in our population-based sample. Self-reported glaucoma was not associated with depression or anxiety. A lack of a burden of depressive symptoms may result from recruitment from a population-based sample as compared to previous study groups predominantly recruited from tertiary care hospitals.

## Background

Glaucoma is a chronic, progressive eye disease, which is characterized by optic nerve damage and visual field loss [1]. It is the second leading cause of irreversible blindness worldwide [2], with approximately 8 million cases of bilateral blindness in 2010 [3]. The number of people with glaucoma was estimated to be 64 million in 2013 [4], increasing to 80 million by 2020 [3]. Treatment often includes the use of multiple eye drops and surgery, however, optic nerve damage cannot be reversed and many patients present disease progression despite therapy. The fear of potential vision loss may result in a higher prevalence of depression and anxiety among glaucoma patients compared to the general population. Depression is a frequent comorbid condition observed in chronic diseases [5, 6] and consequently it has been debated in the past years, whether depression is increased in glaucoma patients or not. Several studies showed a higher prevalence of depressive symptoms among glaucoma patients [7–11], while Wilson et al. did not confirm this finding [12]. The prevalence of depression in subjects with glaucoma has been estimated to be between 10 and 32% in previous studies [7, 11].

Anxiety frequently co-occurs with depression, and anxiety disorders may rise in subjects with glaucoma due to fear of potential blindness and restrictions in everyday activities [13]. Several community-based studies analyzed the prevalence of depression [8, 14] in glaucoma patients, but so far, to the best of our knowledge, this is the first study analyzing both depression and anxiety in a European cohort.

The Gutenberg Health Study (GHS) is a population-based, prospective cohort study in Germany. It includes physical examinations, interviews and questionnaires, related to physical and mental health conditions. This database can be used to determine the prevalence of depression and anxiety among subjects with self-reported glaucoma, and to identify associated factors modifying possible relationships between self-reported glaucoma and psychological disturbances. The results of this study can be used to improve understanding and awareness of mental diseases in glaucoma patients.

## Methods

The Gutenberg Health Study (GHS) is an ongoing population-based, interdisciplinary, prospective, observational single-center cohort study in the Rhein-Main Region in western mid-Germany. It was approved by the ethics committee (Ethics Commission of the State Chamber of Physicians of Rhineland-Palatinate, reference no. 837.020.07, original vote: 22.3.2007, latest update: 20.10.2015). According to the Declaration of Helsinki, written informed consent was obtained from all subjects before entering the study. The population sample was randomly drawn via local residents’ registration offices and equally stratified by sex and residence (urban/rural) for each decade of age. Exclusion criteria were physical and mental disability to visit the study center on their own and an insufficient knowledge of the German language. A detailed description of the study design has been published elsewhere [15].

### Study sample

The baseline sample of 15,010 participants aged 35 to 74 years of the Gutenberg Health Study (GHS) was analyzed in this study.

### Materials and assessment

The baseline-examination was carried out between 2007 and 2012, lasted 5 h and included evaluation of prevalent classical cardiovascular risk factors and clinical variables, laboratory examinations from a venous blood sample, blood pressure and anthropometric measurements, as well as ophthalmological examinations and computer-assisted personal interviews [16].

### Ophthalmological examinations

The ophthalmic part was described in detail by Höhn et al. [16]. In brief, the ophthalmic examinations included objective refraction and distance-corrected visual acuity (DCVA) (Humphrey Automated Refractor / Keratometer [HARK] 599; Carl Zeiss Meditec AG, Jena, Germany) and intraocular pressure (IOP) (Nidek NT-2000; Nidek, Co., Gamagori, Japan).

Self-reported eye diseases and history of any form of glaucoma surgery were assessed by questions during the eye examination. Participants were asked if they suffer from glaucoma, cataract, macular degeneration, corneal diseases or diabetic retinopathy. Additionally, participants were asked if they used any eye drops. Antiglaucoma eye drops were classified using the Anatomic Therapeutic Chemical (ATC) code, including following substance groups: Sympathomimetics (S01EA), Parasympathomimetics (S01 EB), Carbonic anhydrase inhibitors (S01EC), Beta-blockers (S01ED), Prostaglandins (S01EE).

### Questionnaires

Depression was assessed with the Patient Health Questionnaire (PHQ-9), which evaluates how often subjects have been bothered by any of the nine diagnostic criteria of Major depression over the last two weeks. The total points for each of the items were summed up to create a score between 0 and 27 points. Depression was defined as a sum score of ≥10. A prior study showed a sensitivity of 81% and a specificity of 82% for any depressive disorder [17]. In addition, depressive symptoms were classified as “minimal” (score 5 to 9), “mild” (score 10 to 14), “moderately severe” (score 15 to 19) and “severe” (score > 20) [18]. Generalized anxiety was measured with the two screening items of the short form of the GAD-7 (Generalized Anxiety Disorder [GAD]-7 Scale), a screening tool for all anxiety disorders. Participants rated “Feeling nervous, anxious or on edge” and “Not being able to stop or control worrying” by 0 = “not at all”, 1 = “several days”, 2 = “over half the days”, and 3 = “nearly every day”. Generalized anxiety (GAD-2) was defined as a sum score of ≥3, corresponding to a sensitivity of 86% and a specificity of 83% [19].

### Computer-assisted personal interview

During the computer-assisted personal interview subjects were asked if they had the history of any depressive or anxiety disorder. According to Lampert and Kroll, the socioeconomic status (SES) was defined by education, income and job position, with a range from 3 to 21, while 3 indicated the lowest SES and 21 the highest SES [20]. For statistical analyses, three groups were defined including low SES (3–8 points), medium SES (9–14 points) and high SES (15–21 points) [21].Furthermore, subjects were asked to classify their general health condition into four categories (Excellent = 1, good = 2, fair = 3 and poor = 4), and the following self-reported general diseases were collected from the personal interview: arterial hypertension, myocardial infarction, stroke, diabetes mellitus, chronic obstructive pulmonary disease (COPD) and cancer.

### Statistical analysis

Descriptive analyses were performed as absolute and relative proportions for categorical data, mean and standard deviation for continuous variables with approximately normal distribution and median with interquartile range if not fulfilling this criterion.

Prevalence rates of depression and depressive symptoms, resp. generalized anxiety were given for subjects with and without self-reported glaucoma in our study sample.

To validate self-reported glaucoma data, we conducted a sensitivity analysis and included only those self-reported glaucoma subjects, who reported the use of antiglaucoma drugs or having history of glaucoma surgery. Furthermore, a subgroup analysis was performed, to evaluate only glaucoma-subjects with advanced disease. Prevalence of depression and anxiety was computed for subjects with and without self-reported glaucoma and a visual acuity >0.5 logMar in the worse eye. Comparisons between groups were done with chi-squared-test for categorical variables and with Mann-Whitney U-test and t-test for continuous variables.

To determine a relationship between self-reported glaucoma and depression (caseness: PHQ-9 sum score < 10 vs. PHQ-9 sum score ≥ 10), resp. generalized anxiety (caseness: GAD-2 sum score < 3 vs. GAD-2 sum score ≥ 3) we performed logistic regression analysis with depression (PHQ-9 sum score) resp. anxiety (GAD-2 sum score) as the dependent variable. In model 1, self-reported glaucoma was included as independent variable, adjusted for age, sex and socio-economic status. In the following models, we additionally adjusted stepwise for systemic comorbidities (arterial hypertension, myocardial infarction, stroke, diabetes mellitus, chronic obstructive pulmonary disease, cancer), ocular diseases (cataract, macular degeneration, corneal diseases, diabetic retinopathy), visual acuity of the worse eye, IOP, antiglaucoma eye drops (Sympathomimetics, Parasympathomimetics, Carbonic anhydrase inhibitors, Beta-blockers, Prostaglandins) and general health status. The last model included all the above mentioned independent variables.

All p-values should be regarded as a continuous parameter that reflect the level of statistical evidence and are therefore reported exactly. Statistical analysis was carried out using R version 3.3.1 [22].

## Results

14,657 (98% of 15,010) subjects were analyzed and 293 (2.0%) reported having glaucoma. Characteristics of the total study sample and participants with and without self-reported glaucoma are described in Table 1.

**Table 1:** Characteristics and general health condition of participants with and without self-reported glaucoma in the Gutenberg Health Study (GHS), 2007–2012

	All (*N* = 14,657)	No self-reported glaucoma (*N* = 14,364) mean or %	Self-reported glaucoma (*N* = 293) mean or %	*P* value
Demographics				
Age, years	55	55	66	<0.0001
Sex (women) (%)	49.5	49.5	51.2	0.6
SES	12.0	12.0	11.0	0.0001
High (3–8) (%)	18.3	18.1	25.0	0.004
Medium (9–14) (%)	46.2	46.2	48.6	0.4
Low (15–21) (%)	5.5	35.7	26.4	0.001
Comorbidities and general health status				
Diabetes mellitus (%)	9.3	9.2	17.1	<0.0001
Arterial hypertension (%)	49.6	49.1	71.7	<0.0001
Myocardial infarction (%)	2.9	2.9	3.8	0.38
Stroke (%)	1.9	1.9	2.4	0.51
COPD (%)	5.0	4.9	8.5	0.01
Bronchial asthma (%)	2.9	2.8	5.5	0.01
Cancer (%)	9.9	8.9	13.7	0.01
General health status	2.11 ± 0.64	2.10 ± 0.64	2.18 ± 0.61	0.03
Excellent (%)	12.7	12.8	7.5	0.01
Good (%)	66.9	66.8	70.2	0.23
Fair (%)	17.4	17.4	18.5	0.64
Poor (%)	12.7	12.8	7.5	0.01

*SES* socioeconomic status, *COPD* chronic obstructive pulmonary disease

The mean age of subjects with glaucoma was 11 years older compared to the non-glaucoma group. Subjects with glaucoma presented more often with a low SES compared to the non-glaucoma group. General comorbidities such as diabetes mellitus, arterial hypertension, stroke, COPD, bronchial asthma and cancer were more often seen in the glaucoma-group. For ocular comorbidities, the distributions were similar in both groups except for cataract. Mean visual acuity in the worse eye was relatively good for both groups (0.1 vs. 0 logMar). Ophthalmological data are presented in Table 2.

**Table 2:** Ocular characteristics of participants with and without self-reported glaucoma in the Gutenberg Health Study (GHS), 2007–2012

	All (*N* = 14,657)	No self-reported glaucoma (*N* = 14,364) mean or %	Self-reported glaucoma (*N* = 293) mean or %	*P* value
logMar DCVA	0.05	0	0.1	<0.0001
IOP (mmHg)	14	14	15.75	0.0001
Ocular diseases				
AMD (%)	0.5	0.5	1.4	0.05
Corneal diseases (%)	2.0	2.0	3.8	0.05
Cataract (%)	30.7	30.3	49.5	<0.0001
Diabetic retinopathy (%)	0.5	0.5	0.7	0.66
Antiglaucoma drugs				
Sympathomimetics (%)	0.3	0.0	11.6	<0.0001
Parasympathomimetics (%)	0.0	0.0	1.4	<0.0001
Carbonic anhydrase inhibitors (%)	0.3	0.1	12.6	<0.0001
Beta-blockers (%)	1.4	0.2	61.1	<0.0001
Prostaglandins (%)	0.7	0.0	30.4	<0.0001

*IOP* intraocular pressure, *DCVA* distantce-corrected visual acuity

There was no difference in the prevalence of depression between subjects with and without self-reported glaucoma. The prevalence of depression was 6.6% (95% CI 4.1–10.3) for participants with self-reported glaucoma compared to 7.7% (95% CI 7.3–8.2) for participants without self-reported glaucoma (p = 0.58).

For both groups, the prevalence of severe depression was very low compared to minimal depressive symptoms and the distribution of depression severity was comparable (Table 3). Of the participants with self-reported glaucoma 69.1% presented no depressive symptoms (vs. 64.8%; p = 0.14), 24.3% minimal (vs. 27.5%; *p* = 0.26), 4.9% mild (vs. 5.8%; *p* = 0.61), 1.7% moderately severe (vs. 1.5%; *p* = 0.62) and 0.0% severe (vs. 0.4%; *p* = 0.63) depressive symptoms.

**Table 3:** Prevalence of anxiety and depression and severity of depressive symptoms in participants with and without self-reported glaucoma in the Gutenberg Health Study (GHS), 2007–2012

	All (*N* = 14,657)	No self-reported glaucoma (*N* = 14,364) mean or %	Self-reported glaucoma (*N* = 293) mean or %	*P*-value
Depression				
PHQ-9 ≥ 10 (%)	7.7	7.7	6.6	0.58
History of depression (%)	12.0	12.1	9.6	0.24
None (%)	64.9	64.8	69.1	0.14
Minimal (%)	27.4	27.5	24.3	0.26
Mild (%)	5.8	5.8	4.9	0.61
Moderately (%)	1.5	1.5	1.7	0.62
Severe (%)	0.4	0.4	0.0	0.63
Anxiety				
GAD-2 ≥ 3 (%)	6.6	6.6	5.3	0.47
History of Anxiety (%)	7.3	7.3	6.5	0.65

*PHQ-9* Patient Health Questionnaire, *GAD-2* Generalized Anxiety Disorder-2 Scale

With respect to anxiety, prevalence did not differ between the glaucoma and non-glaucoma group. Prevalence for anxiety was 5.3% (95% CI 3.1–8.7) in the glaucoma group vs. 6.6% (95% CI 6.2–7.1) in the non-glaucoma group (*p* = 0.47) (Table 3).

To validate self-reported glaucoma data, we analyzed glaucoma defined as self-reported glaucoma and the use of antiglaucoma eye medications or history of glaucoma surgery. According to this definition, 333 subjects had glaucoma. Comparable prevalence of anxiety (5.3% in the glaucoma group vs. 6.6% in the non-glaucoma group, *p* = 0.37) and of depression (7.0% vs. 7.7% in the non-glaucoma group, *p* = 0.75) was found (Table 4).

**Table 4:** Sensitivity Analysis of participants with new glaucoma definition: Self-reported glaucoma + antiglaucoma drugs and/or history of glaucoma surgery

	All (*N* = 14,657)	No glaucoma (*N* = 14,324) mean or %	Glaucoma (*N* = 333) mean or %	*P*-value
Depression				
PHQ-9 ≥ 10 (%)	7.7	7.7	7.0	0.75
History of depression (%)	12.0	12.0	10.6	0.49
None (%)	64.9	64.8	69.1	0.11
Minimal (%)	27.4	27.5	23.9	0.15
Mild (%)	5.8	5.8	5.2	0.72
Moderately (%)	1.5	1.5	1.8	0.48
Severe (%)	0.4	0.4	0.0	0.64
Anxiety				
GAD-2 ≥ 3 (%)	6.6	6.6	5.3	0.37
History of Anxiety (%)	7.3	7.3	5.7	0.34

*PHQ-9* Patient Health Questionnaire, *GAD-2* Generalized Anxiety Disorder Scale

To identify subjects with an advanced disease, we performed a subsample analysis between those having a visual acuity >0.5 logMar in the worse eye. Only 4.1% (597) of the sample had a visual acuity >0.5 logMar. In the glaucoma group 8.9% had a visual acuity >0.5% compared with 4.0% in the non-glaucoma group. Both prevalence of depression and anxiety were higher for the non-glaucoma group in this subsample analysis (Table 5). Prevalence for depression in glaucoma and non-glaucoma subjects with VA logMar >0.5 were 0 and 8.7% (*p* = 0.25). Prevalence for anxiety in glaucoma and non-glaucoma subjects with VA logMar >0.5 were 4.2 and 5.4% (*p* = 1.0).

**Table 5:** Subsample Analysis of participants with logMar BCVA>0.5 in the worse eye

	All (*N* = 597)	Self-reported no glaucoma (*N* = 571) mean or %	Self-reported glaucoma (*N* = 26) mean or %	*P*-value
Depression				
PHQ-9 ≥ 10 (%)	8.3	8.7	0.0	0.25
History of depression (%)	13.6	14.3	0.0	0.04
None (%)	59.5	59.2	66.7	0.53
Minimal (%)	32.1	32.1	33.3	1.00
Mild (%)	6.7	7.1	0.0	0.40
Moderately (%)	1.4	1.5	0.0	1.00
Severe (%)	0.2	0.2	0.0	1.00
Anxiety				
GAD-2 ≥ 3 (%)	5.4	5.4	4.2	1.00
History of Anxiety (%)	9.1	9.5	0.0	0.16

*PHQ-9* Patient Health Questionnaire, *GAD-2* Generalized Anxiety Disorder Scale

To investigate associations between self-reported glaucoma and depression, resp. anxiety we performed logistic regression analyses. After adjusting for age, sex and SES we found no association between self-reported glaucoma and depression (OR 1.08, 95% CI 0.69–1.62), resp. anxiety (OR 1.15, 95% CI 0.7–1.78). After additional adjustment for general comorbidities (namely arterial hypertension, diabetes mellitus, myocardial infarction, stroke, COPD, cancer), visual acuity (worse eye), IOP, ocular diseases (namely cataract, AMD, corneal diseases, diabetic retinopathy), antiglaucoma drugs (namely eye drops categorized into sympathomimetics, carbonic anhydrase inhibitors, beta-blockers, prostaglandins) and general health status we also found no association (Table 6).

**Table 6:** Associations of self-reported glaucoma with depression and anxiety in the Gutenberg Health Study (GHS), 2007–2012

	Model 1				Model 2			
	OR	CI	*P*-Value	n	OR	CI	*P*-Value	n
Depression (PHQ-9 ≥ 10)	1.08	0.69–1.62	0.72	14,359	1.10	0.50–2.38	0.80	14,061
Anxiety (GAD-2 ≥ 3)	1.15	0.70–1.78	0.55	14,288	1.48	0.63–3.30	0.35	13,991

*PHQ-9* Patient Health Questionnaire, *GAD-2* Generalized Anxiety Disorder Scale, *OR* Odds ratio, *CI* 95% confidence Interval

Model 1: logistic regression analysis adjusted for age, sex, socio-economic status; Model 2 additionally adjusted for systemic comorbidities (arterial hypertension, myocardial infarction, stroke, diabetes mellitus, chronic obstructive pulmonary disease, cancer), ocular diseases (cataract, macular degeneration, corneal diseases, diabetic retinopathy), visual acuity of the worse eye, IOP, antiglaucoma eye medications (Sympathomimetics, Parasympathomimetics, Carbonic anhydrase inhibitors, Beta-blockers, Prostaglandins) and general health status

## Discussion

This is the first population-based study in a large European cohort analyzing the association between a) self-reported glaucoma and depression and b) self-reported glaucoma and anxiety.

The present study did not show a higher prevalence of depression or anxiety in self-reported glaucoma subjects compared to control subjects and there was no statistically significant association between glaucoma and depression or anxiety.

In our study sample the prevalence for depression was 6.6% in the glaucoma group and 7.7% in the non-glaucoma group. The prevalence for anxiety was 5.3% in the glaucoma group and 6.6% in the non-glaucoma group.

Prior studies showed a great inconsistency in depression prevalences among subjects with glaucoma. Depression prevalences varied between 4.3 and 32.1% [7, 8, 10–12, 14, 23–25]. Anxiety prevalences among glaucoma patients were reported to be as high as 13.0% in Japan [7], 22.9% in China [25] and 64% in Singapore [10]. However, it should be noted that these studies differed in study design, sample size and study population. For example, Lim et al. [10] showed depression and anxiety prevalence of 30 and 64%, which is much higher than in our study and compared to other studies. This study was conducted as a case-control study with the study population recruited from tertiary care hospitals. Those studies with subjects recruited from a tertiary care hospital seem to have higher prevalence rates in general which may be an overestimation due to increased severity of cases. Glaucoma patients treated in hospitals present more often disease progression and complex medical records and consequently may have a higher risk for depression or generalized anxiety. Most of the participants of the GHS probably did not require medical treatment in a tertiary care hospital due to their glaucoma disease. Population-based studies have a more heterogeneous study sample, representative of the general population. Prevalence for depression in two other population based studies were 4.3% [14] and 10.9% [8]. It should be considered that an underestimation in a population based study is possible, since severe depression may prevent subjects from attending such studies. Regarding the distribution of depression classes in the current study, there is a low prevalence of moderately severe and severe depression in both groups. In the glaucoma and non-glaucoma group moderately severe depression was seen in 1.7 and 1.5% (*p* = 0.62) and severe depression in only 0.4% of the non-glaucoma group (*p* = 0.63). A recent cross-sectional study evaluating 1520 patients for glaucoma status, dry eye, mood and sleep disorders showed similar depression and anxiety rates in the glaucoma group receiving prostaglandin monotherapy and the non-glaucoma group. These findings are consistent with our results. The authors assume that social awareness of glaucoma and advanced diagnostics tools have led to an improvement in disease management and may contribute to the prevention of psychiatric disorders in glaucoma patients [26].

Our study provides a wide range of socio-demographic factors and comorbidities to assess a possible independent association between glaucoma and depression or anxiety.

In both groups the prevalence of self-reported cataract is high, with 49.5% in the glaucoma group and 30.3% in the non-glaucoma group and might be a confounding factor. Population-based studies showed that depressive symptoms and anxiety were associated with self-reported cataract [14, 27]. However adjusting for ophthalmological diseases including cataract did not alter our findings.

We conducted multiple regression analyses to evaluate a potential relationship. In none of the models could we find an association between glaucoma and depression or anxiety. This is consistent with the results of Wang et al., who also did not observe a significant association after including self-reported general health condition in the regression model. Glaucoma was also self-reported in this population based study [8]. Wilson et al. enrolled a prospective case-control study and stated that self-reported glaucoma patients are not more depressed than patients without glaucoma [12]. The survey instrument used for identifying depression was different from ours, in contrast to the study of Wang et al., where also the PHQ-9 was applied. Both are established survey instruments with a high reliability and validity and useful screening tests for identifying symptoms of depression [17, 28]. The results of Wang et al. might suggest that the perception of having poor general health, induced by an eye disease may be the causal link between eye disease and depression. Consistent with their findings, objective measures of glaucoma such as visual acuity and visual field defects were not associated with depression, in contrast to self-reported measures of visual disability. Self-reported measures were determined from a validated instrument for self-reporting visual disability (the National Eye Institute Visual Function Questionnaire). Su et al. also demonstrated a high impact of systemic comorbidities on depression rates among glaucoma patients in a case-control study. They compared glaucoma patients with a diagnosis of depression to age- and sex-matched glaucoma control subjects without depression. A significantly higher percentage of subjects with a high comorbidity index score was found in the depression group (*p* < 0.0001) [24]. Other previous studies showed that self-reported measures like General Health Status, rather than objective parameters were predictors of depression in glaucoma [8, 11].

The percentage of ß-blockers use is relatively high (61.1%) in our study. The use of systemic ß-blockers was proposed to have neuropsychiatric side effects and to be a possible risk factor for depression [29]. Therefore, some clinicians presume that glaucoma patients may have a higher risk of depression due to the side effects of topically applied ß-blockers [30]. Prior studies could not find the use of topical β-blockers to be associated with depression or anxiety in the multivariate logistic regression model [8, 10, 12, 24]. Presumably the systemic concentration of topically applied ß-blockers is not sufficient to contribute to psychiatric side effects.

The strength of this present study is the population-based design, with a large sample size, standardized examinations and a broad variety of potential confounders. Furthermore, we used established survey instruments with high reliability and validity to identify depression and general anxiety. However, our study also has some limitations. Due to the population-based study design the sample size of the glaucoma group is small, compared to the control group. Glaucoma diagnosis was self-reported, which may lead to recall-error. In our study design optical coherence tomography (OCT) was not included. OCT is an imaging technology that can be used for visualizing and analysing retinal tissue quickly and noninvasively [31]. It enables the detection of structural loss in the retinal nerve fiber layer (RNFL) before perimetric visible visual field defects and has become standard of care for detection of glaucoma and monitoring its progression and for detecting macular and retinal diseases [32]. However, we diminished the recall error of self-reported glaucoma diagnosis by analyzing a subsample, which included only self-reported glaucoma subjects with an additional use of antiglaucoma medications or having a history of glaucoma surgery. This subsample analysis revealed similar results. Additional examination of glaucoma subjects with a visual acuity >0.5 (logMar) in the worse eye again led to the same results. Generally, the number of subjects with reduced visual acuity in our study was low. Only 4.1% (597) of the total sample had a visual acuity >0.5 in the worse eye. Subjects with severe visual impairment might not participate in such a study. This study also comprised only a small number of participants with severe depressive symptoms. A reason could be that severe depression prevents subjects from participating in studies. Finally, the study did not include quantitative visual field measurements to categorize glaucoma severity. A categorization by glaucoma severity might reveal different depression and anxiety prevalence in different glaucoma stages. However, a previous study indicated that the worse eye’s visual acuity was a stronger predictor of depression than the mean deviation, implying that central visual impairment may be more likely to contribute to depression than peripheral visual loss [11].

## Conclusions

We did not find participants with self-reported glaucoma to be more depressed or anxious than participants without self-reported glaucoma. Adjustment for potential confounders did not alter our findings substantially. Future studies are needed to analyze the risk of mental disorders in glaucoma of different severities.

## Abbreviations

AMD: Age-related macular degeneration
ATC: Anatomic therapeutic chemical code
COPD: Chronic obstructive pulmonary disease
DCVA: Distance-corrected visual acuity
GAD: Generalized anxiety disorder scale
GHS: The Gutenberg Health Study
IOP: Intraocular pressure
PHQ: Patient health questionnaire
SES: Socioeconomic status
